# “European” race-specific metacentrics in East Siberian common shrews (*Sorex araneus*): a description of two new chromosomal races, Irkutsk and Zima

**DOI:** 10.3897/CompCytogen.v11i4.19800

**Published:** 2017-11-24

**Authors:** Svetlana V. Pavlova, Sergei A. Borisov, Alexander F. Timoshenko, Boris I. Sheftel

**Affiliations:** 1 A.N. Severtsov Institute of Ecology and Evolution, Russian Academy of Sciences, 33 Leninsky pr., 119071 Moscow, Russia; 2 Irkutsk Antiplague Research Institute of Rospotrebnadzor, 78 Trilissera str., 664047 Irkutsk, Russia; 3 Center of Hygiene and Epidemiology in Irkutsk Region, 51 Trilissera str., 664047 Irkutsk, Russia

**Keywords:** karyotype, chromosomal race, Robertsonian translocation, *Sorex araneus*, Eastern Siberia

## Abstract

Karyotype studies of common shrews in the vicinity of Lake Baikal (Irkutsk Region, Eastern Siberia) resulted in the description of two new chromosomal races of *Sorex araneus* Linnaeus, 1758 (Lypotyphla, Mammalia), additional to 5 races formerly found in Siberia. In the karyotypes of 12 specimens from 3 locations, the polymorphism of metacentric and acrocentric chromosomes of the Robertsonian type was recorded and two distinct groups of karyotypes interpreted as the chromosomal races were revealed. They are geographically distant and described under the racial names Irkutsk (Ir) and Zima (Zi). Karyotypes of both races were characterized by species-specific (the same for all 74 races known so far) metacentric autosomes *af, bc, tu* and *jl*, and the typical sex chromosome system – XX/XY_1_Y_2_. The race-specific arm chromosome combinations include three metacentrics and four acrocentrics in the Irkutsk race (*gk, hi, nq, m, o, p, r*) and four metacentrics and two acrocentrics in the Zima race (*gm, hi, ko, nq, p, r*). Within the races, individuals with polymorphic chromosomes were detected (*g/m, k/o, n/q, p/r*). The presence of the specific metacentric *gk* allowed us to include the Irkutsk race into the Siberian Karyotypic Group (SKG), distributed in surrounding regions. The Zima race karyotype contained two metacentrics, *gm* and *ko*, which have been never found in the Siberian part of the species range, but appear as the common feature of chromosomal races belonging to the West European Karyotypic Group (WEKG). Moreover, the metacentrics of that karyotype are almost identical to the Åkarp race (except the heterozygous pair *p/r*) locally found in the southern Sweden. One of two Siberian races described here for the first time, the Zima race, occurs in an area considerably distant from Europe and shares the common metacentrics (*gm, hi, ko*) with races included in WEKG. This fact may support a hypothesis of independent formation of identical arm chromosome combinations due to occurrence of the same centric fusions in different parts of the species range.

## Introduction

The common shrew *Sorex araneus* Linnaeus, 1758 (Soricidae, Lypotyphla, Mammalia) is a small insectivore mammal and one of large-sized species of the genus. The species is distributed across northern Eurasia from British Isles up to the south-eastern part of Yakutia (Eastern Siberia, Russia) (Zaitsev et al. 2014). Since the 1970s, karyotype polytypy of the species has been known and dozens chromosomal races (currently 74 races known) have been described in different parts of the species range (Shchipanov and Pavlova 2016) according with the rules of the International *Sorex araneus* Cytogenetic Committee, ISACC (Hausser et al. 1994). However, there are still “white spots” where karyotypic status of common shrews remains to be unknown, for instance, the northern and eastern parts of the species range.

Chromosomal differences in polymorphic common shrew result from centric (Rb) fusions of two acrocentric chromosomes into a bi-armed metacentric or alternatively, fissions of a metacentric into two acrocentrics. Ten acrocentric chromosomes (*g, h, i, k, m, n, o, p, q, r*) are involved in both Rb translocations and WART, and different combinations of those acrocentrics cause current karyotypic diversity in the species.

It has been found that the distribution of races with similar karyotypes does not seem to be random, most likely due to common ancestry, and usually neighbor races share one or more of the same metacentrics. Initially, only three phylogenetic groups had been described: West European, East European and Siberian (Searle 1984; Wójcik 1993; Ivanitskaya 1994). Afterwards, 49 known races have been combined into four main karyotypic groups: the West (WEKG), East (EEKG) and North (NEKG) European, and Siberian (SKG) (Searle and Wójcik 1998). The existence of a separate group of races, the NEKG, was first supposed by Fredga (1987). Later, there was an assumption about the relationship between the Scandinavian and Siberian races (Halkka et al. 1994), and this was confirmed by the findings of new chromosomal races in the Ural Region (Polyakov et al. 1997; 2000b). It is very important to note that although all 10 race-specific chromosomes were used for the cladistic analysis, the combinations including only three large arms – *g, h*, and *i* were responsible for the separation of races into individual karyotypic groups (Searle and Wójcik 1998).

It has recently been shown that two centers of high karyotypic diversity of races occur in Russia (Shchipanov and Pavlova 2017). One center is located near the border of Last Glacial Maximum glacier (near the Baltic Sea) while the other one lies near Lake Baikal in Eastern Siberia. Despite less karyotypic data being available from the second center in comparison with the Baltic area, the level of chromosomal variation was found to be significant in both cases. Nevertheless, it is obvious that new data on karyotypic diversity from the easternmost part of the common shrew range will allow us to provide more comprehensive comparative analysis.

In this paper we present data on karyotypic variation of shrews collected from a so far unstudied area in Eastern Siberia (Russia), and discuss a hypothesis whether chromosomal translocations result in the appearing of the same arm combinations in geographically remote races independently.

## Material and methods

Common shrews were collected at three localities in the south-eastern part of the Irkutsk Region, Eastern Siberia, Russia: 1) 17 km SW of the Zima city on the left bank of the River Oka; 2) 17 km E of the Irkutsk city (23^th^ km of the Goloustnoe tract) on the left bank of the River Angara; 3) 5 km SE of the Bayanday village on the left bank of the River Angara. Capture locations were determined using a GPS (Garmin) personal navigation system. A total of 22 common shrews were trapped by home-made live-traps (Shchipanov 1986; Shchipanov et al. 2008) in July-August 2016; karyotypes were obtained from 12 individuals (Table 1).

**Table 1.** New karyotypic data on common shrews from Eastern Siberia (only race-specific chromosomes indicated). Polymorphism for Rb translocation is indicated by slash (/). 2*n*A – diploid number of autosomes.

Site	Locality name	Lat/Lon	Number of specimen, sex	2*n*A	Karyotype	Race
**Irkutsk Region**						
1	*Zima*	53°51'10", 101°49'27"	2 f, 1 m	20	*gm, hi, ko, nq, p, r*	ZIMA
			1 m	20	*gm, hi, ko, n/q, p/r*	
			1 m	21	*gm, hi, ko, n/q, p, r*	
			1 f	21	*gm, hi, k/o, nq, p, r*	
			1 m	21	*g/m, hi, ko, nq, p, r*	
			1 f	22	*g/m, hi, ko, n/q, p, r*	
2	*Irkutsk*	52°17'24", 104°41'54"	3 m	22	*gk, hi, nq, m, o, p, r*	IRKUTSK
3	*Bayanday*	52°59'31", 105°40'10"	1 f	23	*gk, hi, n/q, m, o, p, r*	IRKUTSK

Mitotic chromosome preparations were made in the field from the bone marrow and/or spleen after colchicine treatment *in vivo* following generally Ford and Hamerton (1956) method with some modifications. Briefly, animals were injected intraperitoneally with 0.25 ml of 0.04% colchicine solution for 1-1.5 hours, then cells were washed out in a 10-ml vial by warm phosphate-buffered saline (PBS, Paneco, Russia) using a 2-ml injection syringe; incubated with 5 ml of 0.56% KCl solution for 20 min at 37°C; and fixed with freshly prepared cold glacial acetic-methanol (1:3) for 30 min then twice for 10 min. The cells are concentrated for each change of reagent by centrifuging for 5 min at 100 g.

The trypsin–Giemsa staining technique of Král and Radjabli (1974) was used for identification of each chromosome arm by G-bands. The racial status of each individual was determined according to the standard nomenclature for the karyotype of the common shrew (Searle et al. 1991) and described in terms of the metacentrics (e.g. *pr*), free acrocentrics (*p, r*), or heterozygous state (*p/r*) in the variable autosomal arms *g* to *r.* The nucleolus organiser regions (NORs) were detected by silver nitrate staining following Graphodatsky and Radjabli (1988).

## Results

**Figure 1.**
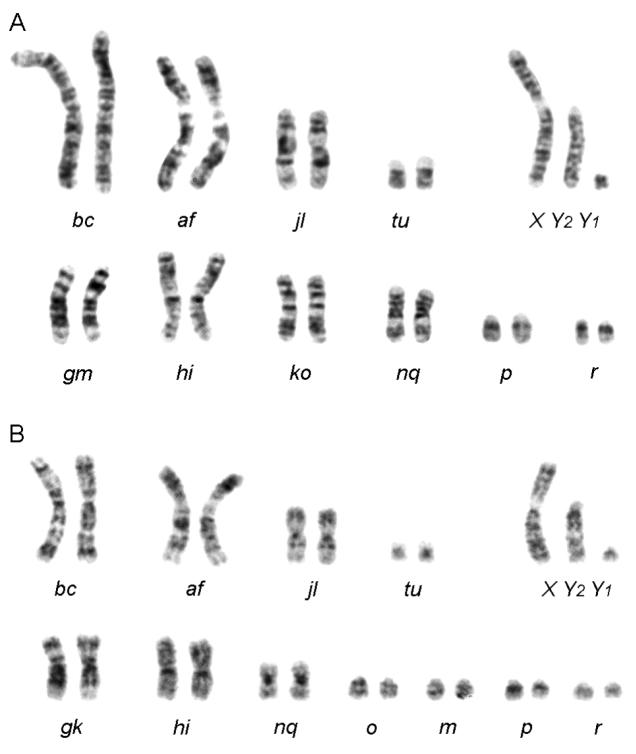
G-banded karyotypes of *S. araneus* males of the Zima 2n=23, FNa=20 (**A**) and Irkutsk 2n=25, FNa=22 (**B**) races. XY_2_Y_1_ – sex chromosomes.

All studied individuals had karyotypes typical for the common shrew (Table 1). The two large (*af, bc*), one medium (*jl*) and one small (*tu*) biarmed pairs of autosomes are invariant and the same in all known chromosomal races over the species range (Fig. 1). The typical sex chromosome system, XX and XY_1_Y_2_ in females and males, respectively, was found in all karyotypes. The diploid number (2n) differs between specimens due to different sex chromosomes and possible polymorphism in the autosome complement.

Two different types of race-specific autosome sets (the variable part of a karyotype) were determined using G-banding: shrews from site 1 had the arm combination of metacentrics *gm, hi, ko, nq* and two acrocentrics *p* and *r*, whereas all other individuals (sites 2, 3) were characterized by three metacentrics *gk, hi, nq* only and four acrocentrics *m, o, p* and *r* (Table 1). Only six of 12 individuals examined had homozygous karyotypes (three in site 1 and three in site 3), while the others were Rb heterozygotes, i.e., they had one or two polymorphic metacentrics and *n/q* variant was most frequent. No Rb heterozygous karyotypes found among the shrews from the site 2.

Silver nitrate staining was applied to confirm the G-banding results of chromosome arms *m* and *o*; the localization of NORs was revealed on the chromosome arm *o* in the metacentric *ko* as well as at the terminal ends of the chromosome arms *q*, *t* and *u*.

Following the rules of the standard nomenclature for *S. araneus* karyotype proposed by Hausser et al. (1994), we give the description of two new chromosomal races of the common shrew (*Sorex araneus*):

**Zima race** (Zi). XX/XY_1_Y_2_, *af, bc, g/m, hi, jl, k/o, n/q, p/r, tu*.

**Type locality.** Zima city and railway station vic., Irkutsk Region, Eastern Siberia, Russia, 53°51'N, 101°49'E.

**Distribution.** Type locality only.

**Additional information.** The same karyotype as the Åkarp race except chromosome arms *p* and *r* presented mostly as free acrocentrics in the Zima race (a single individual with *p/r* was found).

**Irkutsk race** (Ir). XX/XY_1_Y_2_, *af, bc, gk, hi, jl, n/q, m, o, p, r, tu*.

**Type locality.** Irkutsk city vic., Irkutsk Region, Eastern Siberia, Russia, 52°17'N, 104°41'E.

**Distribution.** Known from two sites. The range presumably is located in the south-eastern part of Irkutsk Region.

## Discussion

Among all 12 analyzed shrews we determined two main karyotypic variants which differ by combination of Rb metacentrics – *gm* and *ko*, and *gk*.

Despite some polymorphic metacentrics determined among shrew karyotypes from the vicinity of Zima city (site 1) (Table 1), the arm combination of the first variant (*gm, hi, ko, nq, p/r*) is unique and the sample represents a new chromosomal race. Almost the same karyotype has been previously recorded in the Åkarp race (*gm, hi, ko, nq, pr*) distributed in southern Sweden (Fredga and Nawrin 1977). Contrary to homozygous karyotypes of the Åkarp race, almost all studied shrews from Siberia were heterozygotes (*g/m, k/o, n/q, p/r*). Also, all karyotypes, except a single individual, contained the chromosomes *p* and *r* as acrocentrics, while the Åkarp race had metacentric *pr.* Thus, in our sampling the only stable metacentric pair was *hi.* According with the rules of ISACC (Hausser et al. 1994), the range of the Åkarp race is so remote from the area where the same chromosome arm combination of race-specific metacentrics has been found that we are able to consider studied karyotypes as a new race. The race is titled “Zima” following the name of the nearest city and railway station.

The second karyotypic variant (*gk, hi, nq, m, o, p* and *r*) was determined among shrews collected from south-easternmost part of Irkutsk Region (the southwestern bank of Lake Baikal). Except a single individual from site 3 (Table 1) with polymorphic pair *n/q*, all other karyotypes were homozygous. Because this autosomal arm constitution is different from any other known *S. araneus* karyotypes, we describe this population as another new chromosomal race named after the nearest big city of Irkutsk.

Until recently, five chromosomal races (except the now invalid Altai race, Polyakov et al. 2003) have been discovered in easternmost part of species range (Eastern Siberia) – Tomsk, Ilga, Yermakovskoie, Strelka and Baikal (Polyakov et al. 2000a; Sheftel et al. 2016). Of the races, the Baikal and Strelka races have the fewest race-specific metacentrics, *hi* and *go* and *hi*, respectively. Other three races are characterized the metacentric *gk* which is a marker of the Siberian karyotypic group (SKG), thus, the Irkutsk race (*gk* and *hi*) certainly belongs to the group.

Regarding the Zima race (*gm, hi* and *ko*), the picture is more complicated because *gm* and *hi* metacentrics mark the races of the West European Karyotypic group (WEKG). The most eastern European races carrying metacentric *gm* are the Mologa and Penza races and the Kirillov race which are distributed on the right bank alongside the River Volga and the River Mezen, respectively (up to a longitude of 50°). The metacentric *hi* has been found in the race-specific karyotypes belonging to both the WEKG and Siberian group; however, it has been suggested that the metacentric could have originated independently in each of such spatially remote groups (Searle and Wójcik 1998). Thus, beyond the European part of the species range, we identified the *gm* and *ko* metacentrics in *S. araneus* karyotypes for the first time.

There are some cases when a chromosomal race inhabits an area beyond the main range of a group. For example, all neighbors of the Neroosa race (*go, hi*) distributed in European Russia belonged to another group (WEKG). The same picture can be found for the Strelka race that has the metacentric *go* but all surrounding races belong to the SKG (except the Zima race with *gm* and *hi*). In all these cases, the isolated races are distributed close to the range of its own group, i.e. do not distant more than a range of a single race. So, we may suppose that the races within a karyotypic group have origins related to other races of a group but current isolation of ranges may be explained by hybridization or/and an impact of environmental factors.

In other cases a chromosomal race has a distribution significantly distant from the area occupied by related races of the same karyotypic group. As an example, the Bergen race inhabits the western part of Norway; while two other races having the same metacentric are distributed in Finland and the western Russia (Kalvitsa and Lemi). Similarly, the races carrying metacentric *gr* occupy an area along the southern bank of Baltic Sea, whereas recently discovered the Poyakonda race, *Py* (Pavlova 2010) inhabits the south territory of the Kola peninsula (White Sea, Russia). In both cases, it may be logical to assume the independent origin of the same metacentric variant (*gr*).

Diagnostic metacentrics *gm* and *hi* mark the WEKG, but the type locality of new Zima race is located more than 3 thousand kilometers away from the area of European races. Thus, the metacentric *gm* in the Zima race could have appeared independently during karyotypic evolution. According with the rules of the ISACC, two races having the same karyotype (the case of the Zima and Åkarp races) but isolated by distance should be considered as two different races.

**Figure 2.**
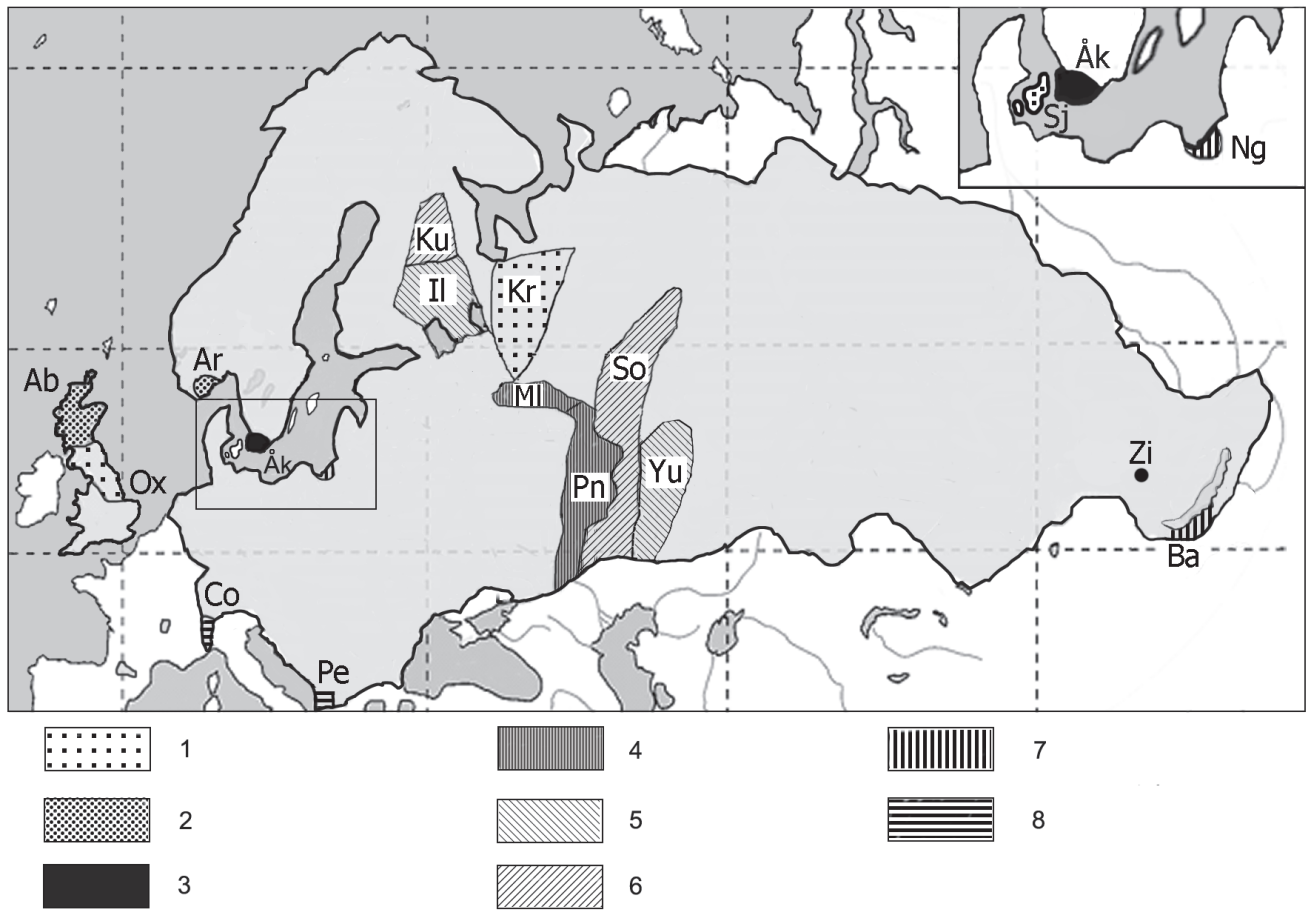
The distribution of chromosomal races of the common shrew sharing the same race-specific chromosomes in a karyotype (differ only by the presence of polymorphic metacentrics in some individuals): **1** Oxford (Ox) – Sjaelland (Sj) – Kirillov (Kr) **2** Aberdeen (Ab) – Arendal (Ar) **3** Åkarp (Åk) – Zima (Zi) **4** Mologa (Ml) – Penza (Pn) **5** Ilomantsi (Il) – Yuryuzan (Yu) **6** Kuhmo (Ku) – Sok (So) **7** Nogat (Ng) – Baikal (Ba) **8** Pelister (Pe) – Cordon (Co).

There are examples when two or even three races have the same set of race-specific chromosomes and here we list some of them (Table 2; Fig. 2). It is worth to note that the first five races (cases 1 and 2) belong to the WEKG, while other four races (cases 5 and 6) – to the North European karyotypic group. It has been suggested that range expansion of a single chromosomal race in the past is more likely explanation of the current karyotypic identity of the Oxford, Sjaelland and Kirillov races than the assumption that five new metacentrics could have arose independently in karyotypes of those three races due to the same Rb fusions (Shchipanov and Pavlova 2016). In the case of the Aberdeen and Arendal races, we may assume similar scenario of the range expansion from the south of Scandinavia to the south of Britain, whereas Polyakov with co-authors (2000b) suggested that the Ilomantsi - Yuryuzan, and the Kuhmo - Sok races have independent evolutionary origin.

**Table 2.** Some examples of identical set of race-specific metacentrics and acrocentrics in different chromosomal races of the common shrew.

No in Fig. 2	Race names	Distribution	Karyotype
1	Oxford Sjaelland Kirillov	England Denmark European Russia	*g/m, hi, k/q, no, p/r*
2	Aberdeen Arendal	England Sweden	*gm, hi, ko, np, q/r*
3	Åkarp** Zima	Sweden Eastern Siberia	*g/m, hi, k/o, nq, p/r*
4	Mologa and Penza	European Russia	*g/m, hn, i/o, kr, p/q*
5	Ilomantsi Yuryuzan	N European Russia Ural mountains	*g/o, hn, i/p, k/r, m/q*
6	Kuhmo Sok	Finland European Russia	*g/o, hn, i/p, k/g, m/r*
7	Nogat Baikal	Poland Eastern Siberia	*g, hi, k, m, n, o, p, q, r*
8	Pelister Cordon	Macedonia Switzerland	*g, h, i, k, m, n, o, p, q, r*

Here we mention the pair of the Mologa - Penza races (case 4) that have the same karyotypes. However, it should be noted that there is a probability of incorrect description of the Penza race because their karyotypes differ by the presence of polymorphism *g/m* in Penza race only and the fact of isolation of two ranges is still unclear (Orlov et al. 2007).

Also, several “acrocentric” races share identical race-specific chromosomes (cases 7 and 8), but they are occur in areas very far from each other.

The type localities of chromosomal races Åkarp and Zima (case 3) are located in very remote parts of the species range (at a distance of more than 6000 km from each other), and so it is hard to explain its current location as a result of dispersion of a single chromosomal race in the past. Moreover, in contrast to the Zima race, all studied karyotypes from Sweden were homozygous and completely metacentric. Taking into account high level of polymorphic metacentrics in the sampling from the type locality of the Zima race, it might be supposed possible ways of chromosomal evolution of studied races by accumulation of Rb translocations or WARTs. For example, a single WART is required to create the Zima race from the Strelka race or several Rb fusions in the Baikal race karyotype might also have resulted in the Zima race. Because only metacentric *hi* was found to be fixed in the studied karyotypes, we can also hypothesize that the Zima population might originate after hybridizing between the Baikal race and hypothetical full metacentric race with *gm, hi, ko, nq* and *pr*.

There is one more example of similar to the case of the Zima race. The Istranca race distributed in European Turkey has arm combination *ik*, which was not recorded in geographically close populations from south-eastern, central or western Europe but found in races distributed in north-eastern Europe and Siberia.

Thus, these examples allow us to assume the possibility of independent formation of identical chromosome arm combinations due to centric translocations in racial karyotypes of the common shrew.
